# Clinical status of patients 1 year after hospital discharge following recovery from COVID-19: a prospective cohort study

**DOI:** 10.1186/s13613-022-01034-4

**Published:** 2022-07-10

**Authors:** Dapeng Li, Xuejiao Liao, Zhenghua Ma, Lina Zhang, Jingke Dong, Guoqin Zheng, Mei Zi, Wujian Peng, Lanlan Wei, Zhiyan Li, Yingjun Kong, Lifei Wang, Dongjing Liu, Fang Wang, Qing He, Guobao Li, Zheng Zhang, Lei Liu

**Affiliations:** 1 Institute for Hepatology, National Clinical Research Center for Infectious Disease, Shenzhen Third People’s Hospital; The Second Affiliated Hospital, School of Medicine, Southern University of Science and Technology, No. 29, Bulan Road, Longgang district, Shenzhen, 518112 China; 2 Department of Chronic Disease Follow-Up, Shenzhen Third People’s Hospital, No. 29, Bulan Road, Longgang district, Shenzhen, 518112 China; 3 Department of Respiratory Medicine, Shenzhen Third People’s Hospital, No. 29, Bulan Road, Longgang district, Shenzhen, 518112 China; 4 Department of Nephrology, Shenzhen Third People’s Hospital, No. 29, Bulan Road, Longgang district, Shenzhen, 518112 China; 5 Department of Ultrasound, Shenzhen Third People’s Hospital, No. 29, Bulan Road, Longgang district, Shenzhen, 518112 China; 6 Department of Radiology, Shenzhen Third People’s Hospital, No. 29, Bulan Road, Longgang district, Shenzhen, 518112 China; 7 Department of the Third Pulmonary Disease, Shenzhen Third People’s Hospital, No. 29, Bulan Road, Longgang district, Shenzhen, 518112 China; 8 Shenzhen Research Center for Communicable Disease Diagnosis and Treatment of Chinese Academy of Medical Science, No. 29, Bulan Road, Longgang district, Shenzhen, 518112 China; 9 Guangdong Key Laboratory for Anti-Infection Drug Quality Evaluation, No. 29, Bulan Road, Longgang district, Shenzhen, 518112 China

**Keywords:** COVID-19, Pulmonary dysfunction, Extrapulmonary dysfunction, SARS-CoV-2 antibodies, Risk factor

## Abstract

**Background:**

The long-term clinical status of coronavirus disease 2019 (COVID-19) in recovered patients remains largely unknown**.** This prospective cohort study evaluated clinical status of COVID-19 and explored the associated risk factors.

**Methods:**

At the outpatient visit, patients underwent routine blood tests, physical examinations, pulmonary function tests, 6-min walk test, high-resolution computed tomography (CT) of the chest, and extrapulmonary organ function tests.

**Results:**

230 patients were analyzed. Half (52.7%) reported at least one symptom, most commonly fatigue (20.3%) and sleep difficulties (15.8%). Anxiety (8.2%), depression (11.3%), post-traumatic symptoms (10.3%), and sleep disorders (26.3%) were also reported. Diffusion impairments were found in 35.4% of the patients. Abnormal chest CT scans were present in 63.5% of the patients, mainly reticulation and ground-glass opacities. Further, a persistent decline in kidney function was observed after discharge. SARS-CoV-2-specific antibodies of IgA, IgG, and IgM were positive in 56.4%, 96.3%, and 15.2% of patients, respectively. Multivariable logistic regression showed that disease severity, age, and sex were closely related to patient recovery.

**Conclusions:**

One year after hospital discharge, patients recovered from COVID-19 continued to experience both pulmonary and extrapulmonary dysfunction. While paying attention to pulmonary manifestations of COVID-19, follow-up studies on extrapulmonary manifestations should be strengthened.

**Supplementary Information:**

The online version contains supplementary material available at 10.1186/s13613-022-01034-4.

## Background

Severe acute respiratory syndrome coronavirus 2 (SARS-CoV-2) is the pathogen causing coronavirus disease 2019 (COVID-19). The COVID-19 epidemic has resulted in huge challenges to global public health and heavy economic and social burdens. As of 29 December 2021, SARS-CoV-2 has infected over 281 million individuals, worldwide, and caused over 5.4 million deaths [1]. Over time, most discharged patients have experienced phases of recovery, but the long-term health effects are still emerging [2].

SARS-CoV-2 mainly invades the lungs during the acute stage, manifesting as pneumonia and acute respiratory distress syndrome [3]. Nevertheless, COVID-19 infections are distinguished by multiple system lesions, leading to multiple extrapulmonary manifestations [4], including thrombotic complications [5], heart injury [6], acute kidney injury [7], gastrointestinal symptoms, liver cell injury, metabolic system abnormalities, and neurologic disease [8]. Previous studies have explored the lung function changes in patients with COVID-19 during the recovery period and identified persistent symptoms [9–11]. Similar long-term health consequences were reported following infections with the related SARS and Middle East respiratory syndrome (MERS) viruses [12]. However, patients with COVID-19 are more likely to demonstrate multiple organ effects, including in the respiratory, endocrine, neural (psychological), and cardiovascular systems [13–19]. However, long-term follow-up studies investigating multiple organ function in recovered patients are lacking.

Here, we report a comprehensive evaluation of the pulmonary and extrapulmonary dysfunction, and the associated risk factors, observed in patients with COVID-19 1 year following their discharge from the hospital.

## Methods

### Study design and participants

This cohort study included patients with COVID-19 admitted to Shenzhen Third People's Hospital, which is the only hospital designated for the treatment of patients with COVID-19 in Shenzhen, Guangdong, China. Between January 11 and April 27, 2020, 462 patients with COVID-19 were hospitalized. Patients were excluded if they were younger than 16 years, died within 1 year of discharge, refused to participate, were lost to contact, and lived outside of Shenzhen city. The research protocol was approved by the Ethics Committee of Shenzhen Third People's Hospital (IRB 2020-021-02). All patients provided written informed consent.

### Data collection and follow-up

Demographic characteristics, laboratory data, and acute-phase medical histories were retrospectively collected from hospital electronic medical records. The acute phase of COVID-19 was defined as the period from symptom onset to hospital discharge. Disease severity was classified into the following categories according to the four severity grades from the clinical guidance for COVID-19 pneumonia diagnosis and treatment, issued by the Chinese National Health Commission: (1) mild illness, patients with mild symptoms and without radiological evidence; (2) moderate illness, patients with fever, respiratory tract symptoms, and radiological evidence of confirmed pneumonia; (3) severe illness, patients with one of the following: (a) respiratory distress (≥ 30 breaths/min); (b) oxygen saturation ≤ 93% at rest; (c) arterial partial pressure of oxygen/fraction of inspired oxygen ≤ 300 mmHg; (4) critical illness, patients with one of the following: (a) respiratory failure requiring mechanical ventilation; (b) shock; (c) other organ failure that requires intensive care unit (ICU) [20, 21]. All discharged patients met uniform discharge standards: no fever for three consecutive days, improved respiratory symptoms, obvious recovery of acute lung lesions, and two negative SARS-CoV-2 test results, 24 h apart. Two months before the start of the follow-up, a nurse contacted each patient, by telephone, and invited them to participate in this study. The patients were contacted in the order of their recorded discharge date. Pulmonary and extrapulmonary functions were evaluated in the outpatient clinic between December 26, 2020 and June 19, 2021.

### General symptom and psychological symptom

Participants completed a 21-item symptom questionnaire to report new and persistent symptoms and any symptoms that were more severe than before COVID-19 onset. Patients participated in face-to-face interviews with an experienced psychologist and were asked to complete four psychological questionnaires: anxiety symptoms were assessed using the Generalized Anxiety Disorder 7-item (GAD-7) scale, depression symptoms using the Patient Health Questionnaire-9 (PHQ-9), post-traumatic stress symptoms (PTSS) using the Post-traumatic Stress Disorder (PTSD) Checklist (PCL-5), and sleep disorders using the Pittsburgh Sleep Quality Index (PSQI). Psychological abnormalities were determined using generally the accepted cut-off values (PHQ-9 ≥ 7, GAD-7 ≥ 7, PCL-5 ≥ 33, and PSQI ≥ 7) [22].

### Pulmonary function test and exercise capacity

Pulmonary function tests were performed using a flow spirometer and the lung diffusing capacity for carbon monoxide (*D*_LCO_) was measured using the single breath method. Pulmonary function parameters included forced vital capacity (FVC), forced expiratory volume in one second (FEV_1_), FEV_1_/FVC, *D*_LCO_, total lung capacity (TLC), and residual volume (RV). Diffusion dysfunction was diagnosed when the *D*_LCO_ was less than 80% of predicted. The 6-min walk test was performed according to the established protocol [23].

### Chest CT scan

High-resolution chest computed tomography (CT) scans were performed with the patient in the supine position at end-inspiration using a uCT 760 scanner (United Imaging, Shanghai, China). The following characteristics of the chest CT scan were recorded: ground-glass opacity (GGO), crazy paving, reticulation, honeycombing, parenchymal bands, consolidation, air trapping, and bronchiectasis. The distribution of pulmonary lesions was described as peripheral, random, or diffuse. To quantify the severity of the lung involvement, a severity score for each lung lobe was determined as the percentage of involvement [24, 25]: no involvement, less than 5% involvement, 5–25% involvement, 26–49% involvement, 50–75% involvement, more than 75%, with corresponding scores of 0, 1, 2, 3, 4, or 5, respectively. The total CT severity score was calculated by summing the scores for all five lung lobes (range, 0–25).

### Extrapulmonary organ function test

Participants underwent a series of extrapulmonary organ function tests, including kidney function tests, serological marker measurements, and ultrasound evaluations of the abdomen and deep veins of the lower limbs. Kidney abnormalities were evaluated using blood and urine laboratory indicators related to renal function, including estimated glomerular filtration rate (eGFR), blood urea nitrogen (BUN), proteinuria, urea α1-microglobulin (A1M), and urea β2-microglobulin (B2M). The calculation of eGFR was based on the Chronic Kidney Disease Epidemiology Collaboration (CKD-EPI) equation. The serological markers related to disease severity determined were the levels of C-reactive protein (CRP), interleukin 6 (IL-6), lactate dehydrogenase (LDH), and D-dimer.

### SARS-CoV-2 antibody test

Plasma samples collected during the acute and follow-up phases were analyzed to assess anti-SARS-CoV-2 total immunoglobulin (i.e., IgA, IgM, and IgG) levels. Commercial enzyme-linked immunosorbent assays (Wantai, Beijing, China), involving magnetic particles coated with receptor-binding domain (RBD) antigens, were performed on the Caris200 automatic chemiluminescence instrument, according to the manufacturer’s protocol. The sample’s cut-off index (COI) = RLU/CO, where RLU is the specimen’s chemiluminescence reaction signal value, and CO is the cut-off value. Specimen’s COI ≥ 1was considered to be positive.

### Statistical analysis

Categorical variables are reported as frequencies and percentages. Continuous variables are expressed as means (standard deviation, SD) or medians (interquartile range, IQR). To compare between-group demographic and clinical variables, Chi-squared tests or Fisher’s exact tests were used for each categorical variable. Student’s *t*-test, Wilcoxon signed-rank test, or analysis of variance was used for continuous variables, as appropriate. Associations between two continuous variables were explored using Pearson or Spearman correlation analyses. Multivariable logistic regression models were used to determine the risk factors associated with the presence of clinical status, and corresponding odds ratios (ORs), with 95% confidence intervals (95% CIs), were calculated by adjusting for age, sex, and disease severity. Statistical significance was defined as a two-sided *P* value < 0.05. Statistical analyses were performed using R (Version 3.5.1).

## Results

### Patient characteristics

**Fig. 1:**
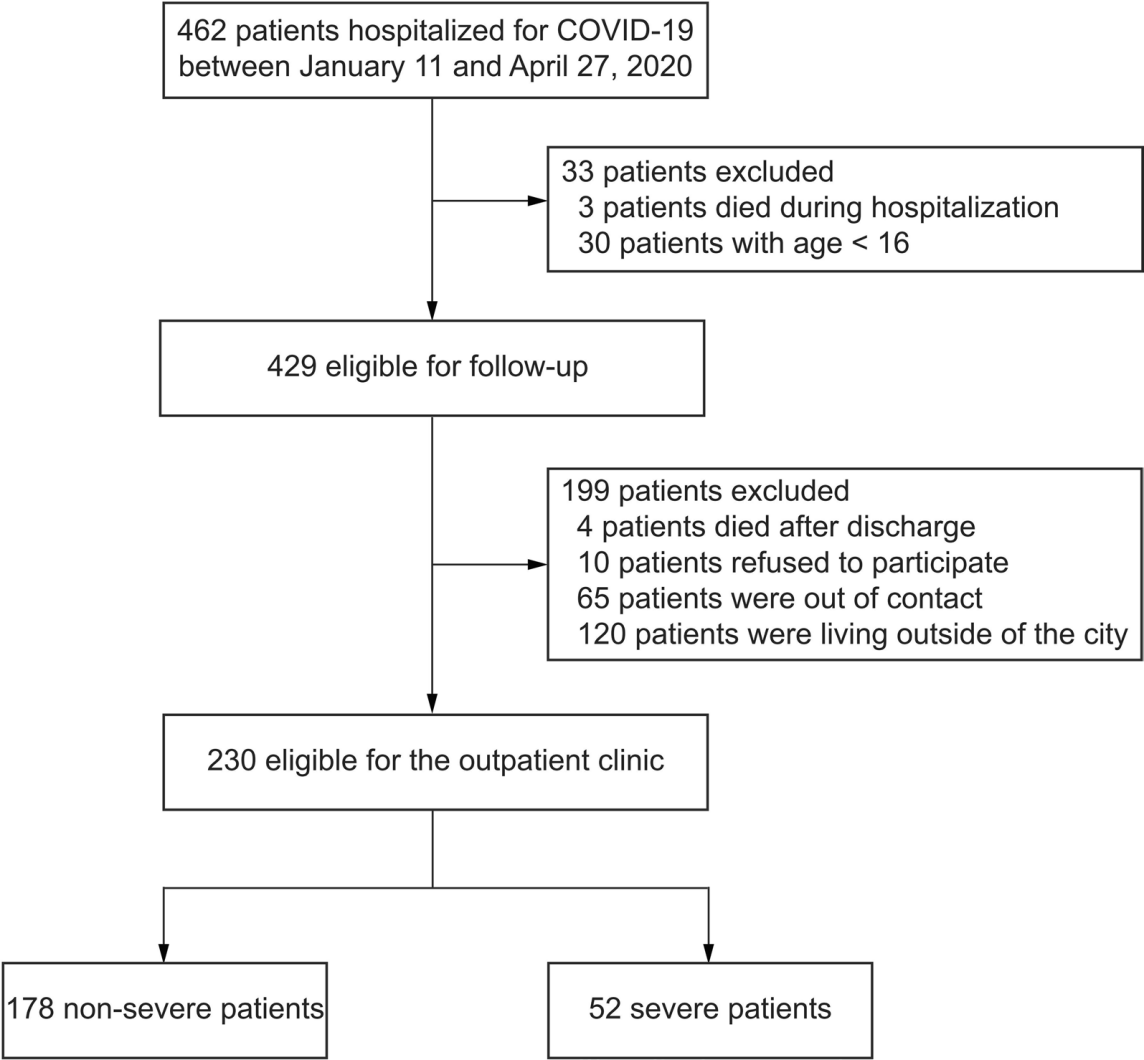
Flowchart diagram of patient enrollment

A total of 230 underwent follow-up assessments in the outpatient clinic between December 26, 2020 and June 19, 2021 (flowchart shown in Fig. 1, Additional file 1: Table S1). Overall, the numbers of included patients with mild, moderate, severe, and critical disease were 12 (5.2%), 166 (72.2%), 45 (19.6%), and 7 (3.0%), respectively. The mean age of the 230 included patients was 46.2 years, and 114 (49.6%) were female. The median follow-up time was 385 days, post-discharge. Comorbidities were present in 50 (21.7%) patients. The median length of hospital stay was 21 days, with 14 (6.1%) patients having severe disease spending time in the ICU (median stay, 12 days). Compared with patients having non-severe disease, patients with severe disease were more likely to be male, older, have longer hospitalization, and have more comorbidities (Table 1).

**Table 1:** Baseline characteristics of included patients with COVID-19

Variables	All patients	Non-severe patients	Severe patients	*P* value
N	230	178	52	
Age, mean (SD), y	46.3 (14.4)	43.79 (13.8)	54.88 (13.2)	** < 0.001**
Sex, female, *N* (%)	114 (49.6)	95 (53.4)	19 (36.5)	**0.048**
Body mass index, mean (SD), kg/m^2^	24.4 (11.4)	24.1 (12.8)	25.3 (3.6)	0.493
Smoking, *N* (%)	19 (8.3)	13 (7.3)	6 (11.5)	0.490
Comorbidities				
Any, *N* (%)	50 (21.7)	31 (17.4)	19 (36.5)	**0.006**
Hypertension, *N* (%)	32 (13.9)	21 (11.8)	11 (21.2)	0.137
Diabetes, *N* (%)	13 (5.7)	6 (3.4)	7 (13.5)	**0.015**
Cardiovascular disease, *N* (%)	6 (2.6)	2 (1.1)	4 (7.7)	**0.034**
Hepatitis B infection, *N* (%)	8 (3.5	6 (3.4)	2 (3.8)	1.000
Cancer, *N* (%)	2 (0.9)	1 (0.6)	1 (1.9)	0.935
Supplementary oxygen required, *N* (%)	155 (67.4)	103 (57.9)	52 (100.0)	** < 0.001**
NIV/IMV required, *N* (%)	29 (12.6)	0 (0.0)	29 (55.8)	** < 0.001**
Hospitalization in ICU, *N* (%)	14 (6.1)	0 (0.0)	14 (26.9)	** < 0.001**
Duration of ICU stay, median (IQR), d	na	na	12 (5.75–19.50)	na
Hospitalization period, median (IQR), d	21 (16–29)	20 (16–22)	30 (21–38)	** < 0.001**

*SD* standard deviation; *IQR* interquartile range; *NIV* non-invasive mechanical ventilation; *IMV* invasive mechanical ventilation; *na* not available

### General symptoms and psychological symptoms

**Fig. 2:**
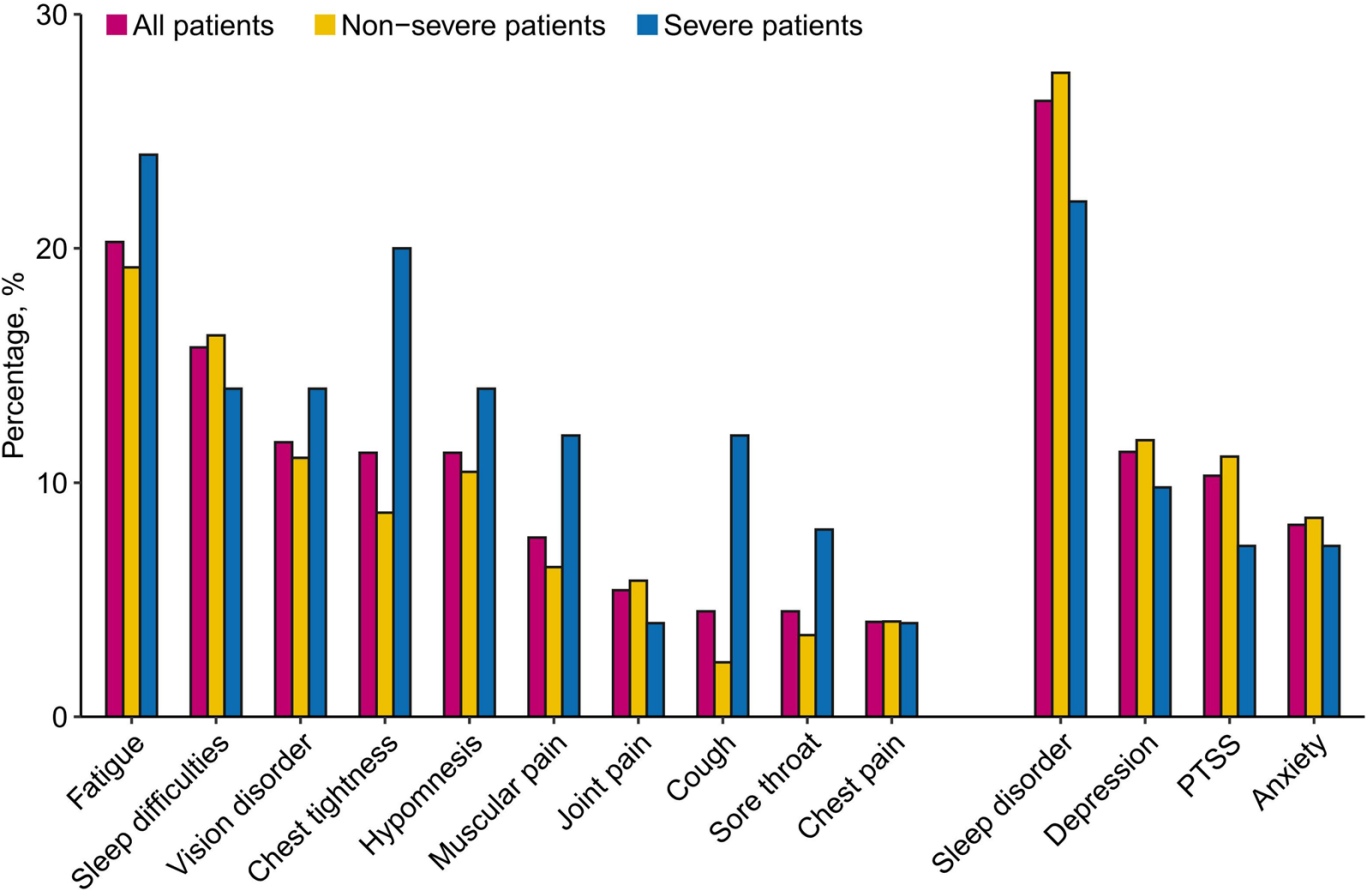
Percentage of ten common general symptoms and psychological symptoms in recovery patients with COVID-19 at 1-year follow-up. PTSS, post-traumatic stress symptoms

General symptoms were assessed in 222 patients at the 1-year follow-up; 52.7% (117 of 222) reported at least one symptom (Additional file 1: Table S2). The most commonly reported symptom was fatigue (20.3%), followed by sleep difficulties (15.8%). Among patients in the severe group, 62.5% of (31 of 50) patients reported ongoing or new symptoms compared with 50.0% (86 of 176) in the non-severe group (Fig. 2). Additionally, chest tightness and cough were significantly more common in patients in the severe group than in those in the non-severe group. Psychological symptoms were evaluated in 194 patients (Additional file 1: Table S2); 70 (36.1%) reported at least one psychological symptom. The prevalences of anxiety, depression, PTSS, and sleep disorders were 8.2%, 11.3%, 10.3%, and 26.3%, respectively, with no statistically significant differences between the non-severe and severe groups.

### Pulmonary assessment

Pulmonary function tests were performed in 113 patients at the 1-year follow-up (Table 2, Additional file 1: Table S3). Diffusion impairment was the most common pulmonary function abnormality, and was 40 (35.4%) patients and 10 (8.8%) had *D*_LCO_ and *D*_LCO_/alveolar volume (*V*_A_) below 80% of predicted values. FVC (3.5% of patients), FEV_1_ (8.0%), TLC (8.0%), and RV (1.8%) abnormalities were also observed. A borderline significant difference in *D*_LCO_ was found between patients in the severe and non-severe group, with a mean value of 86% of predicted in the non-severe group and 80% of predicted in the severe group. Between-group differences were not observed for FEV_1_, FVC, or FEV_1_/FVC; however, there were significant between-group differences in *D*_LCO_/*V*_A_, TLC, and RV. A total of 187 patients completed the 6-min walking test at follow-up, walking a median distance of 478 (IQR, 333–580) meters. The 6-min walking distance for patients in the severe group (415 m) was shorter than that for those in the non-severe group (499 m).

**Table 2:** Pulmonary function test, 6-min walking test, and CT scan at 1-year follow-up

Variables	All patients	Non-severe patients	Severe patients	*P* value *
Pulmonary function, *N*	113	87	26	
FVC < 80% predicted, *N* (%)	4 (3.5)	2 (2.3)	2 (7.7)	0.483
FEV_1_ < 80% predicted, *N* (%)	9 (8.0)	7 (8.0)	2 (7.7)	1.000
FVC/FEV_1_ < 80% predicted, *N* (%)	0 (0.0)	0 (0.0)	0 (0.0)	na
MMEF < 65% predicted, *N* (%)	36 (31.9)	27 (31.0)	9 (34.6)	0.917
*D*_LCO_ < 80%, predicted, *N* (%)	40 (35.4)	27 (31.0)	13 (50.0)	0.123
*D*_LCO_/*V*_A_ < 80%, predicted, *N* (%)	10 (8.8)	5 (5.7)	5 (19.2)	0.084
TLC < 80%, predicted, *N* (%)	9 (8.0)	5 (5.7)	4 (15.4)	0.238
RV < 80% predicted, *N* (%)	2 (1.8)	2 (2.3)	0 (0.0)	1.000
6-min walking test, *N*	187	146	41	
Distance < 450 m, *N* (%)	86 (46.0)	61 (41.8)	25 (61.0)	**0.045**
CT scan, *N*	208	160	48	
Involvement of the lesions, *N* (%)				** < 0.001**
No involvement	76 (36.5)	69 (43.1)	7 (14.6)	
Single lobe	56 (26.9)	48 (30.0)	8 (16.7)	
Bilateral multilobe	76 (36.5)	43 (26.9)	33 (68.8)	
No. of lobes involved, median (IQR)	1 (0 − 2)	1 (0 − 2)	2 (1 − 4)	** < 0.001**
Total CT score, mean (SD)	2.29 (3.29)	1.44 (2.05)	5.15 (4.78)	** < 0.001**

^*^P values were calculated with student *t* test, or Wilcoxon rank sum test, or Chi-squared test

*FVC* forced vital capacity; *FEV*_*1*_ forced expiratory volume in 1 s; MMEF, maximal mid-expiratory flow; *D*_LCO_, diffusing capacity of the lung for carbon monoxide; *D*_LCO_/*V*_A_, *D*_LCO_ corrected for alveolar volume; *TLC* total lung capacity; *RV* residual volume; *na* not available

**Fig. 3:**
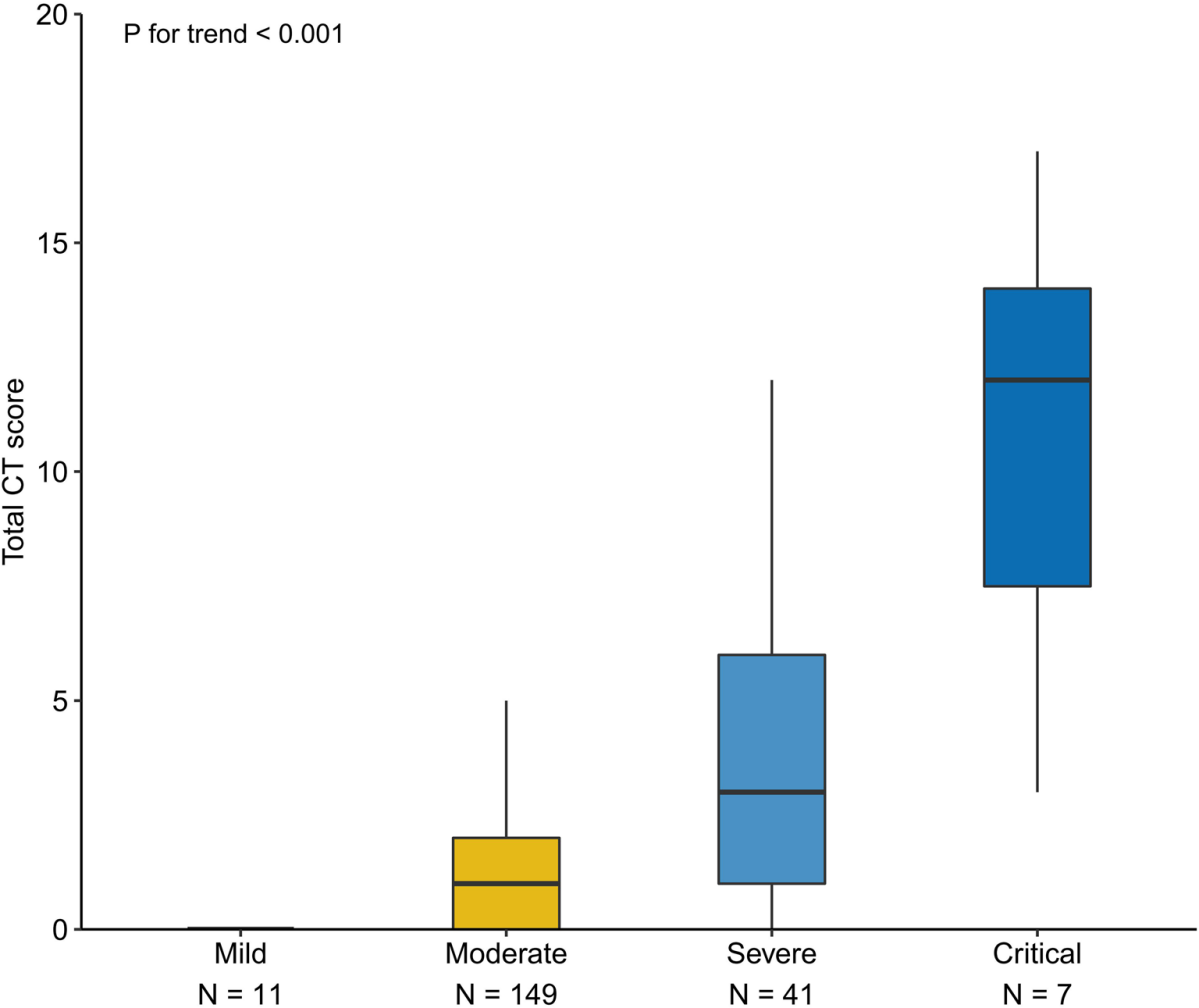
Total CT scores in recovery patients with different disease severity at 1-year follow-up. The trend was determined by linear regression model

Follow-up chest CT scans were performed on 208 patients (Table 2, Additional file 1: Table S3), with pulmonary abnormalities (bilateral involvement and peripheral and diffuse distribution) observed in 132 (63.5%) patients. The most common CT findings were reticulation (40.4%) and GGO (39.4%). The mean total CT score was 2.29 in the patients with severe disease, which was significantly higher than that for patients in the non-severe group. The total CT score showed a linear increase that was observed to correspond with the incremental increase in disease severity (Fig. 3). The total CT score was negatively correlated with pulmonary parameters (Additional file 1: Fig. S1), including FEV_1_/FVC, *D*_LCO_, *D*_LCO_/*V*_A_, and RV. There was no difference in total CT score in patients with *D*_LCO_ < 80% and those with *D*_LCO_ ≥ 80% (3.18 vs 1.85, *P* = 0.056) (Additional file 1: Table S4).

### Extrapulmonary organ assessment

**Fig. 4:**
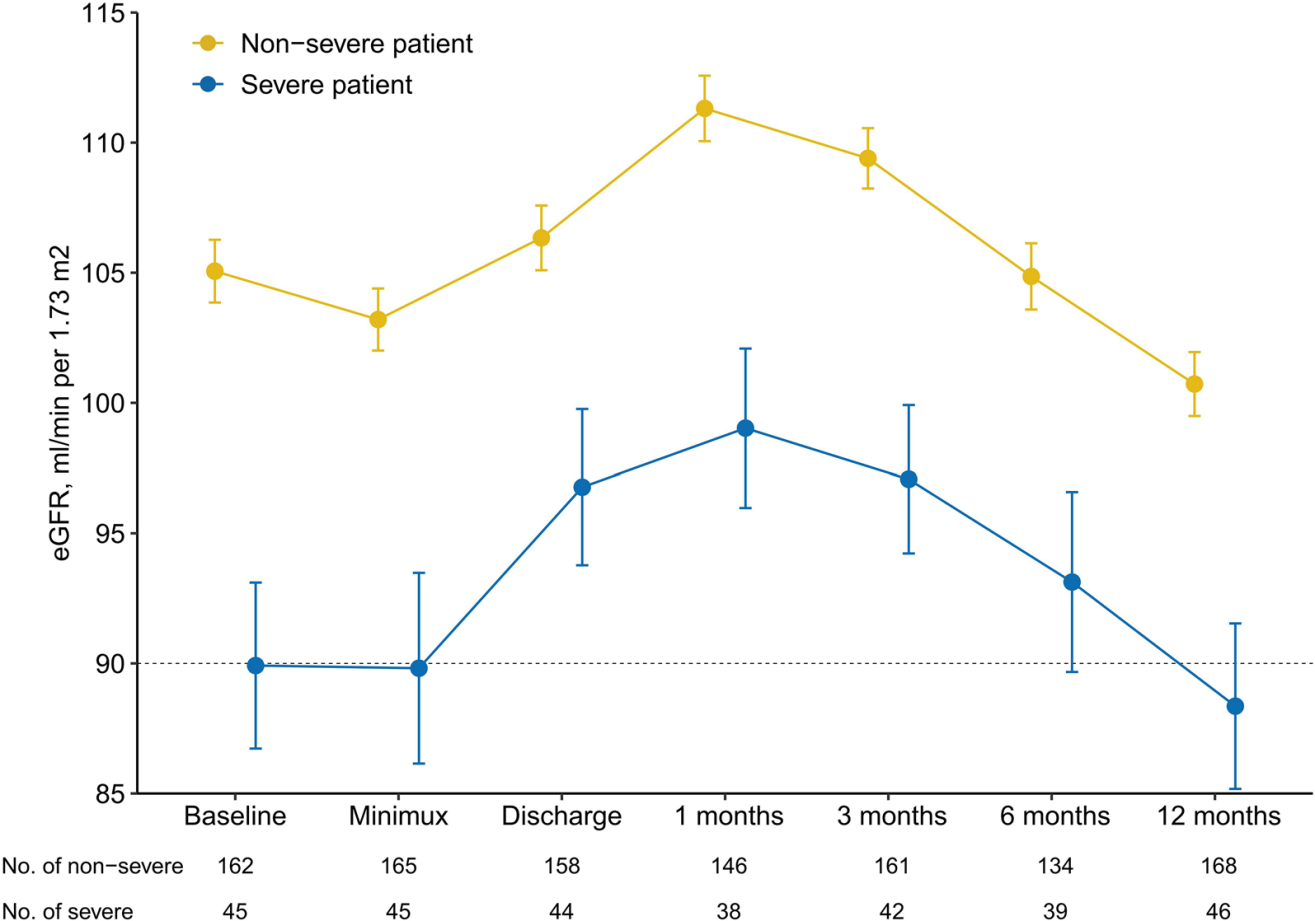
Temporal changes in renal function according to eGFR analysis in recovery patients with different disease severity. Data are represented as mean ± standard error (SE)

Kidney function tests were evaluated in 214 patients (Additional file 1: Table S5). At the 1-year follow-up, 28.5% of patients had decreased eGFR (< 90 mL/min/1.73 m^2^) and fewer patients (19.5%) had proteinuria. A comparison of kidney function parameters between the non-severe and severe patient groups showed a significant difference in the proportions of patients with decreased eGFR (patients in severe group, 45.7%; patients in non-severe group, 23.8%; *P* = 0.006). Kidney function parameters, including BUN, proteinuria frequency, and urea A1M and B2M levels, were significantly elevated in patients in the severe group. A dynamic analysis of eGFR revealed that kidney function decreased from 1 to 6 months follow-up for patients in both the non-severe and severe groups (Fig. 4). Lower limb deep vein thrombosis was not observed in the 106 participants who underwent ultrasound examinations. Biomarkers related to COVID-19 severity, such as CRP, D-dimer, LDH, and IL-6 levels, were higher in patients in the severe group than in those in the non-severe group (Additional file 1: Table S6).

### SARS-CoV-2 antibody

**Fig. 5:**
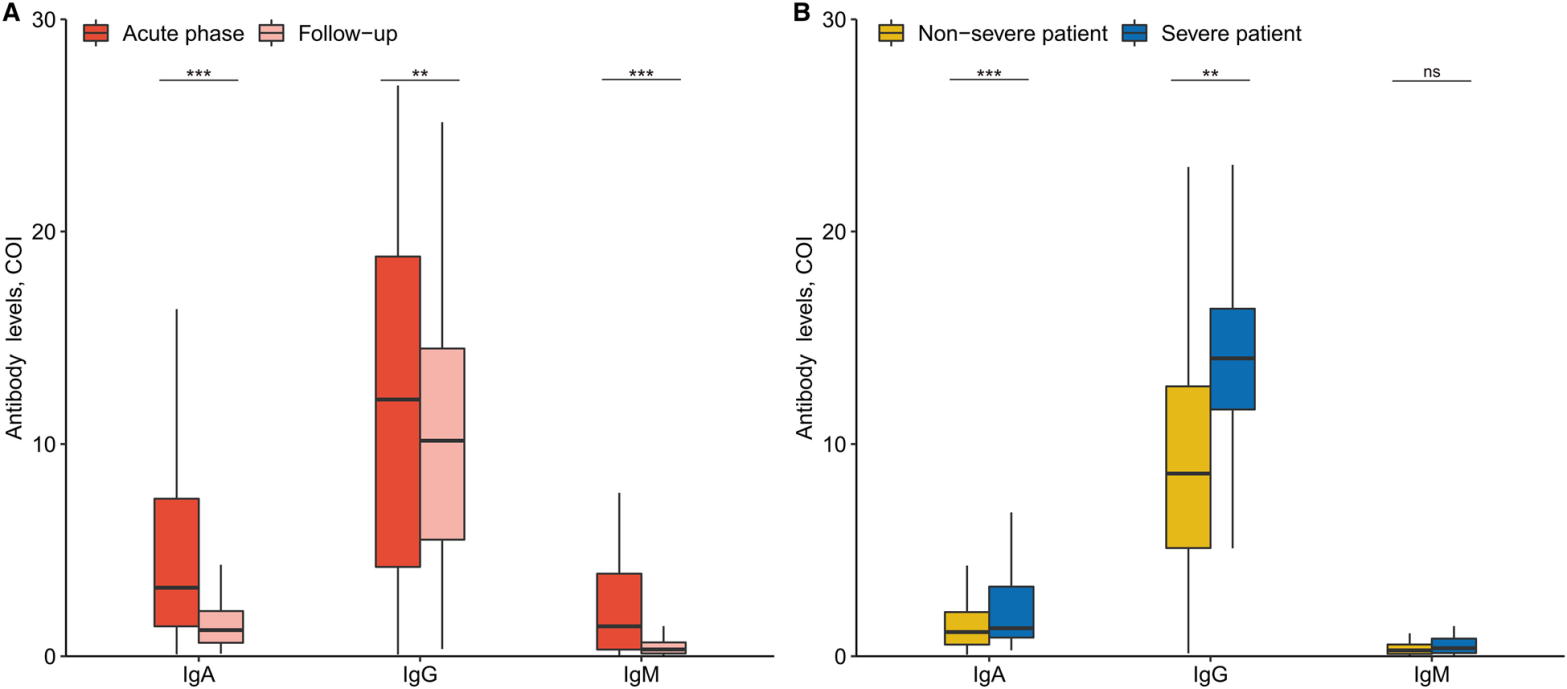
SARS-CoV-2 RBD-specific antibody levels in recovery patients with COVID-19. **A** Levels of anti-RBD IgA, IgG, and IgM antibodies in 163 recovery patients at acute phase and 1-year follow-up. The difference was determined by the paired samples Wilcoxon test. **B** Levels of anti-RBD IgA, IgG, and IgM between 173 non-severe patients and 49 severe patients at 1-year follow-up. The difference between two groups was determined by the Wilcoxon test. COI, cut-off Index. ns, *P* > 0.05; **, *P* ≤ 0.01; ***, *P* ≤ 0.001

SARS-CoV-2 antibody results were obtained for 222 patients at the 1-year follow-up (Additional file 1: Table S7); 126 (56.8%) patients were positive for IgA antibodies, 213 (95.9%) for IgG antibodies, and 34 (15.4%) for IgM antibodies. Among the 222 patients, antibody results from both the acute phase (average detection time, 6.87 days, post-admission) and 1-year follow-up were available for 163 patients. Compared with the acute-phase concentrations, the titers for all three antibody types were significantly decreased at the follow-up assessment (Fig. 5A). The 49 patients in the severe group had significantly higher IgA and IgG levels than did the 173 patients in the non-severe group (Fig. 5B).

### Risk factors associated with health status

Multivariable logistic regression models were applied to assess associations between demographic and clinical factors and health-related status (Additional file 1: Table S8). Age greater than 50 years was positively associated with anxiety, depression, PTSS, and sleep difficulties. Female sex and severe illness were independent risk factors of diffusion impairment. Age and severe illness were associated with radiological abnormalities. Age and male sex were associated with a higher risk of decreased eGFR (< 90 mL/min/1.73 m^2^). Disease severity was a significant indicator of decreased IgG levels (> 50%).

## Discussion

This long-term follow-up study assessed multiple system damage in patients with COVID-19, 1 year after their hospital discharge. We found that more than half of the patients reported at least one symptom. A considerable percentage of patients demonstrated continuing abnormal pulmonary functions, especially diffusion impairment and radiological abnormalities. Moreover, extrapulmonary organ manifestations were also observed in recovered patients, the decline in kidney function is particularly pronounced. SARS-CoV-2-specific IgG antibodies remained positive in most patients.

This study revealed that a wide range of physical and psychological symptoms persist in patients for at least 1 year after being hospitalized due to COVID-19. Specifically, 51% of patients had at least one symptom, with fatigue (20.7%) and sleeping difficulties (15.0%) being the most common. These results are consistent with short-term follow-up studies assessing the prevalence of post-acute COVID-19 syndrome [15, 26, 27]. For example, a prospective cohort study that included patients from Wuhan, China, 6 months after their hospital discharge, demonstrated that 76% of the patients reported at least one symptom; 63% reported fatigue (or muscular weakness) and 26% reported sleep difficulties [2]. Our study found that mental health problems were common among discharged patients at the 1-year follow-up. Similarly, a recent systematic review reported a pooled prevalence of 31% for sleep disturbances, 23% for anxiety, and 12% for depression, and revealed an increased prevalence of psychiatric symptoms between mid- and long-term follow-up [28]. Thus, these studies suggest that discharged patients with psychological symptoms may benefit from early psychological support.

The present study found that a considerable proportion of patients had reduced diffusion capacity at the 1-year follow-up. This was consistent with previous results for patients discharged from hospital for 6 months after recovering from COVID-19, where the prevalence of reduced diffusion capacity was 16–52% [2, 29–31]. A recent study reported that a third of patients demonstrate reduced gas transfer, as measured by *D*_LCO_, 12 months after discharge [9]. Similarly, decreased lung diffusion function was observed in patients who recovered from SARS and MERS infections, after hospitalization or ICU admission, based on the results of a meta-analysis; a pooled prevalence of 24.35% was reported at 6 months [32]. Furthermore, we observed that a considerable proportion of patients demonstrated chest CT scan abnormalities, with reticulation and GGO being most frequent. This is consistent with an earlier study that showed two-thirds of patients having residual radiological abnormalities, predominantly residual GGO and reticulation, 100 days after COVID-19 onset [27].

This study also investigated long-term clinical status in extrapulmonary organs. We and others have shown that angiotensin I converting enzyme 2, the host cell receptor of SARS-CoV-2, is highly expressed in multiple organs, including the lung, heart, kidney, gastrointestinal tract, liver, pancreas, nervous system, and skin [33, 34]. Accordingly, extrapulmonary organ dysfunction have been reported during both the acute and recovery phases [4, 35]. Our kidney function study demonstrated that 28.5% of patients had decreased eGFR (< 90 mL/min/1.73 m^2^), and 18.9% had proteinuria. This is consistent with earlier findings that 35% of patients have decreased eGFR at their 6-month, post-discharge follow-up assessments [2]. Moreover, a trajectory analysis, using our data and those from a previous study, showed that the eGFR continued to decrease after hospital discharge [36]. The decline of eGFR may partly be due to loss of weight explaining the increase in eGFR at 1 months and then patients recovered, gained weight and muscle increasing plasma creatinine and by such lowering eGFR. Because of the lack of kidney assessment before COVID-19 and a follow-up cohort of health controls, the clinical significance of the decline of eGFR after COVID-19 can be further addressed in longer observational studies. The pathogenic mechanism of the decline of the persistent renal function after recovery from COVID-19 is unclear, possibly related to ongoing inflammation, intrinsic tubular injury, maladaptive repair or regaining muscle after discharge. A previous study demonstrated that the SARS-CoV-2 virus can directly infect human renal tubules, causing renal tubule damage and acute kidney injury [37]. According to a urinary microprotein examination, our study found that indicators of renal tubular damage, such as A1M and B2M, were significantly elevated in the patients in the severe group compared with those in the non-severe group. These data suggest that patients who have recovered from COVID-19 may be at risk of persistent renal dysfunction after hospital discharge.

In this study, we reported SARS-CoV-2-specific antibodies in the convalescent serum of patients at the 1-year follow-up assessment, suggesting that anti-SARS-CoV-2 IgG antibodies are detectable in the majority of patients. This finding corresponds with a recent report that indicated that SARS-CoV-2 infection induces humoral immune responses that remain detectable for at least 11 months [38]. Compared with the concentrations during the acute phase, IgG antibodies were significantly decreased at the follow-up visit. Thus, the durability of the IgG antibodies is questionable; however, evidence from another study of SARS-CoV-1 infections showed that IgG antibodies remained detectable for 36 months [39]. Similarly, the seropositivity rate (50.7–56.1%) persisted for up to 4 years after MERS infection, significantly dropping during the 5th year [40]. Our study also showed that the concentrations of IgG antibodies in patients with non-severe disease were significantly lower than in those who had severe disease; the nine patients who had reverted to being IgG-negative for the SARS-CoV-2 antibodies were in the non-severe group. Thus, the decline in antibodies among patients who have recovered from non-severe disease raises concerns of reinfection after repeated exposure to SARS-CoV-2. Continuous monitoring is warranted to confirm the longevity and potency of the anti-SARS-CoV-2 antibody response.

This study has some limitations. First, our study had a single-center design. However, all patients in Shenzhen city with confirmed COVID-19 were admitted to our hospital, facilitating the generalization of these findings. Second, even though some patients were lost to follow-up, there was no significant difference in the clinical characteristics between included and excluded patients. Third, the baseline patient data, such as pulmonary function tests, were unavailable during hospitalization because of preventing cross infection. Fourth, the results should be interpreted with caution because of the missing data of assessments, such as the missing data on pulmonary function tests and the lack of data on dyspnea at follow-up. Finally, a possible limitation was that we cannot rule out that some of them did not attend outpatient clinics, even if all patients diagnosed with COVID-19 in Shenzhen city were required to be evaluated.

## Conclusions

In summary, 1 year after hospital discharge, more than half and one-third of the patients recovering from COVID-19 continued to experience physical and psychological symptoms. Patients continued to experience both pulmonary and extrapulmonary dysfunction. These dysfunctions were more frequent among patients with severe disease.

## Supplementary Information

**Additional file 1:** **Fig S1**. Correlation between total CT score and pulmonary function parameters. **Table S1**. Baseline characteristics of discharged patients with COVID-19 who were followed up compared with who were not. **Table S2**. General symptoms and psychological symptoms in recovery patients with COVID-19 at 1-year follow-up. **Table S3**. Pulmonary function test, 6-min walking test, and CT scan at 1-year follow-up. **Table S4**. Comparison of CT finding according to diffusion capacity of the lung for carbon monoxide. **Table S5**. The kidney function in recovery patients with COVID-19 at 1-year follow-up. **Table S6**. Laboratory biomarkers in recovery patients with COVID-19 at 1-year follow-up. **Table S7**. SARS-CoV-2 RBD-specific antibody levels and seropositive rate in recovery patients with COVID-19 at 1-year follow-up. **Table S8**. Risk factors associated with psychological symptoms, diffusion impairment, radiological abnormalities, decreased eGFR, and decreased IgG.

## Data Availability

The dataset supporting the conclusions of this article is included within the article and its additional file.

## Abbreviations

SARS-CoV-2: Severe acute respiratory syndrome coronavirus 2
COVID-19: Coronavirus disease 2019
MERS: Middle East respiratory syndrome
GAD-7: Generalized Anxiety Disorder 7-item scale
PHQ-9: Patient Health Questionnaire-9
PTSS: Post-traumatic stress symptoms
PCL-5: Post-traumatic Stress Disorder Checklist
PSQI: Pittsburgh Sleep Quality Index
*D*_LCO_: Diffusing capacity for carbon monoxide
FVC: Forced vital capacity
FEV_1_: Forced expiratory volume in one second
TLC: Total lung capacity
RV: Residual volume
GGO: Ground-glass opacity
eGFR: Estimated glomerular filtration rate
BUN: Blood urea nitrogen
A1M: Urea α1-microglobulin
B2M: Urea β2-microglobulin
CRP: C-reactive protein
IL-6: Interleukin 6
LDH: Lactate dehydrogenase
OR: Odds ratio
CI: Confidence interval
