# Supplementary material for: Clinical status of patients 1 year after hospital discharge following recovery from COVID-19: a prospective cohort study
Source: Ann Intensive Care. 2022 Jul 10;12:64. doi: 10.1186/s13613-022-01034-4 (PMC9272871; doi:10.1186/s13613-022-01034-4)
Supplement: Supplementary file 1 — Additional file 1: Fig S1. Correlation between total CT score and pulmonary function parameters. Table S1. Baseline characteristics of discharged patients with COVID-19 who were followed up compared with who were not. Table S2. General symptoms and psychological symptoms in recovery patients with COVID-19 at 1-year follow-up. Table S3. Pulmonary function test, 6-min walking test, and CT scan at 1-year follow-up. Table S4. Comparison of CT finding according to diffusion capacity of the lung for carbon monoxide. Table S5. The kidney function in recovery patients with COVID-19 at 1-year follow-up. Table S6. Laboratory biomarkers in recovery patients with COVID-19 at 1-year follow-up. Table S7. SARS-CoV-2 RBD-specific antibody levels and seropositive rate in recovery patients with COVID-19 at 1-year follow-up. Table S8. Risk factors associated with psychological symptoms, diffusion impairment, radiological abnormalities, decreased eGFR, and decreased IgG. [file 13613_2022_1034_MOESM1_ESM.docx]

**Supplementary Material**
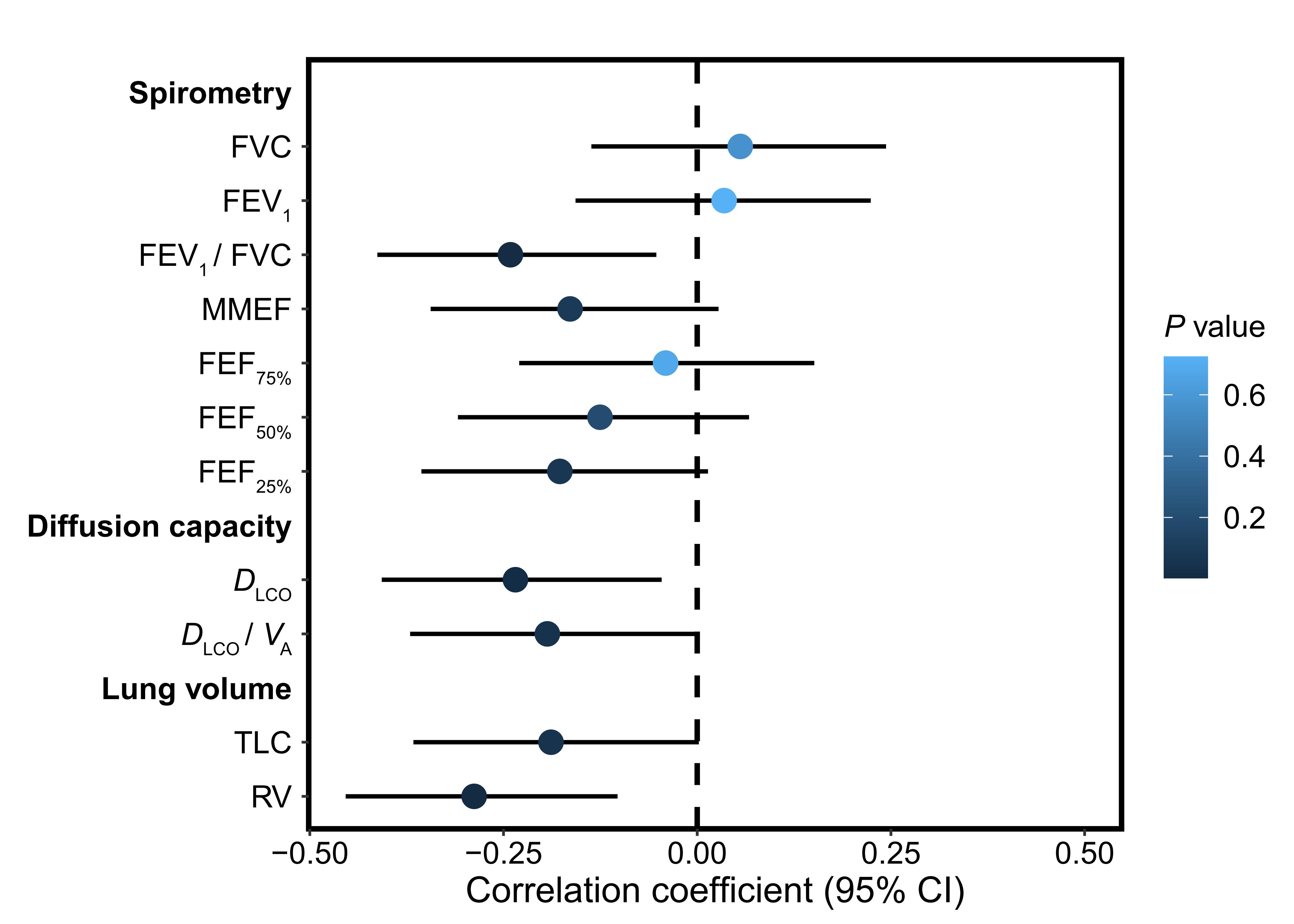


**Fig. S1. Correlation between total CT score and pulmonary function parameters.**

Pearson correlation analysis were applied.

Abbreviations: FVC, forced vital capacity; FEV_1_, forced expiratory volume in 1 s; MMEF, maximal mid-expiratory flow; FEF_75%_, forced expiratory flow at 75% of FVC; FEF_50%_, forced expiratory flow at 50% of FVC; FEF_25%_, forced expiratory flow at 25% of FVC; *D*_LCO_, diffusing capacity of the lung for carbon monoxide; *D*_LCO_/*V* _A_, *D*_LCO_ corrected for alveolar volume; TLC, total lung capacity; RV, residual volume; CI, confidence interval.

**Table S1. Baseline characteristics of discharged patients with COVID-19 who were followed up compared with who were not**

|  | **Overall** | **Participation** | **Nonparticipation** | ***P* value *** |
| --- | --- | --- | --- | --- |
| N | 429 | 230 | 199 |  |
| Age, mean (SD), y | 46.19 (15.45) | 46.30 (14.38) | 46.07 (16.64) | 0.878 |
| Sex, female, N (%) | 213 (49.7) | 114 (49.6) | 99 (49.7) | 1.000 |
| Body mass index, mean (SD), kg/m² | 23.84 (8.65) | 24.35 (11.41) | 23.23 (3.07) | 0.185 |
| Smoking, N (%) | 31 (7.2) | 19 (8.3) | 12 (6.1) | 0.491 |
| Comorbidities |  |  |  |  |
| Any, N (%) | 86 (20.0) | 50 (21.7) | 36 (18.1) | 0.412 |
| Hypertension, N (%) | 54 (12.6) | 32 (13.9) | 22 (11.1) | 0.457 |
| Diabetes, N (%) | 21 (4.9) | 13 (5.7) | 8 (4.0) | 0.578 |
| Cardiovascular disease, N (%) | 13 (3.0) | 6 (2.6) | 7 (3.5) | 0.791 |
| Hepatitis B infection, N (%) | 13 (3.0) | 8 (3.5) | 5 (2.5) | 0.765 |
| Cancer, N (%) | 5 (1.2) | 2 (0.9) | 3 (1.5) | 0.871 |
| Hospitalization in ICU, N (%) | 28 (6.5) | 14 (6.1) | 14 (7.0) | 0.841 |
| Duration of ICU stay, mean (SD), d | 19.04 (13.93) | 15.64 (15.13) | 22.43 (12.22) | 0.203 |
| Hospitalization period, mean (SD), d | 23.10 (9.88) | 23.97 (9.84) | 22.11 (9.85) | 0.051 |
| Disease severity, N (%) |  |  |  | 0.506 |
| Mild | 23 (5.4) | 12 (5.2) | 11 (5.5) |  |
| Moderate | 316 (73.7) | 166 (72.2) | 150 (75.4) |  |
| Severe | 74 (17.2) | 45 (19.6) | 29 (14.6) |  |
| Critical | 16 (3.7) | 7 (3.0) | 9 (4.5) |  |

* *P* values were calculated with student *t* test or Chi-squared test.

**Table S2. General symptoms and psychological symptoms in recovery patients with COVID-19 at one-year follow-up**

| **Symptoms** | **All patients** | **Non-severe patients** | **Severe patients** | ***P* value** * |
| --- | --- | --- | --- | --- |
| **General symptoms, N** | 222 | 172 | 50 |  |
| Any symptom, N (%) | 117 (52.7) | 86 (50.0) | 31 (62.0) | 0.182 |
| Fatigue, N (%) | 45 (20.3) | 33 (19.2) | 12 (24.0) | 0.585 |
| Sleep difficulties, N (%) | 35 (15.8) | 28 (16.3) | 7 (14.0) | 0.866 |
| Vision disorder, N (%) | 26 (11.7) | 19 (11.0) | 7 (14.0) | 0.748 |
| Chest tightness, N (%) | 25 (11.3) | 15 (8.7) | 10 (20.0) | **0.049** |
| Hypomnesis, N (%) | 25 (11.3) | 18 (10.5) | 7 (14.0) | 0.659 |
| Muscular pain, N (%) | 17 (7.7) | 11 (6.4) | 6 (12.0) | 0.313 |
| Joint pain, N (%) | 12 (5.4) | 10 (5.8) | 2 (4.0) | 0.885 |
| Cough, N (%) | 10 (4.5) | 4 (2.3) | 6 (12.0) | **0.012** |
| Sore throat, N (%) | 10 (4.5) | 6 (3.5) | 4 (8.0) | 0.334 |
| Chest pain, N (%) | 9 (4.1) | 7 (4.1) | 2 (4.0) | 1.000 |
| Palpitations, N (%) | 9 (4.1) | 5 (2.9) | 4 (8.0) | 0.230 |
| Smell disorder, N (%) | 8 (3.6) | 8 (4.7) | 0 (0.0) | 0.262 |
| Skin rash, N (%) | 8 (3.6) | 4 (2.3) | 4 (8.0) | 0.143 |
| Sweatiness, N (%) | 7 (3.2) | 3 (1.7) | 4 (8.0) | 0.077 |
| Hair loss, N (%) | 6 (2.7) | 6 (3.5) | 0 (0.0) | 0.399 |
| Chills, N (%) | 5 (2.3) | 4 (2.3) | 1 (2.0) | 1.000 |
| Diarrhoea, N (%) | 4 (1.8) | 2 (1.2) | 2 (4.0) | 0.469 |
| Hearing disorder, N (%) | 2 (0.9) | 1 (0.6) | 1 (2.0) | 0.933 |
| Fever, N (%) | 1 (0.5) | 1 (0.6) | 0 (0.0) | 1.000 |
| Lower extremity edema, N (%) | 1 (0.5) | 0 (0.0) | 1 (2.0) | 0.510 |
| Taste disorder, N (%) | 1 (0.5) | 1 (0.6) | 0 (0.0) | 1.000 |
| **Psychological symptoms, N** | 194 | 153 | 41 |  |
| Any symptom, N (%) | 70 (36.1) | 59 (38.6) | 11 (26.8) | 0.228 |
| Sleep disorders, N (%) | 51 (26.3) | 42 (27.5) | 9 (22.0) | 0.610 |
| Depression, N (%) | 22 (11.3) | 18 (11.8) | 4 (9.8) | 0.934 |
| PTSS, N (%) | 20 (10.3) | 17 (11.1) | 3 (7.3) | 0.674 |
| Anxiety, N (%) | 16 (8.2) | 13 (8.5) | 3 (7.3) | 1.000 |

* *P* values were calculated Chi-squared test.

Abbreviations: PTSS, post-traumatic stress symptoms.

**Table S3. Pulmonary function test, 6-min walking test, and CT scan at one-year follow-up**

| **Variables** | **All patients** | **Non-severe patients** | **Severe patients** | ***P* value *** |
| --- | --- | --- | --- | --- |
| **Pulmonary function, N** | 113 | 87 | 26 |  |
| FVC, mean (SD) | 99(14) | 100(13) | 98(19) | 0.624 |
| FEV_1_, mean (SD) | 96(14) | 96(12) | 96(18) | 0.937 |
| FVC / FEV_1_, mean (SD) | 97 (7) | 97 (7) | 95 (6) | 0.249 |
| MMEF, mean (SD) | 76(21) | 77(21) | 75(22) | 0.761 |
| *D*_LCO_, mean (SD) | 85(14) | 86(14) | 80(13) | 0.066 |
| *D*_LCO_ / *V*_A_, mean (SD) | 96(11) | 97(12) | 90(10) | **0.005** |
| TLC, mean (SD) | 96(11) | 97(11) | 90(10) | **0.006** |
| RV, mean (SD) | 118(22) | 121(22) | 107(14) | **0.003** |
| **6-min walking test, N** | 187 | 146 | 41 |  |
| Distance, m, median (IQR) | 478 (333 - 580) | 499 (342 - 584) | 415 (282 - 530) | 0.085 |
| **CT scan, N** | 208 | 160 | 48 |  |
| Findings, N (%) |  |  |  |  |
| Reticulation | 84 (40.4) | 55 (34.4) | 29 (60.4) | **0.002** |
| GGO | 82 (39.4) | 47 (29.4) | 35 (72.9) | **<0.001** |
| Bronchiectasis | 28 (13.5) | 20 (12.5) | 8 (16.7) | 0.617 |
| Parenchymal band | 16 (7.7) | 13 (8.1) | 3 (6.2) | 0.905 |
| Air trapping | 13 (6.2) | 6 (3.8) | 7 (14.6) | **0.017** |
| Crazy-paving pattern | 12 (5.8) | 2 (1.2) | 10 (20.8) | **<0.001** |
| Consolidation | 7 (3.4) | 6 (3.8) | 1 (2.1) | 0.916 |
| Honeycombing | 2 (1.0) | 0 (0.0) | 2 (4.2) | 0.080 |

* *P* values were calculated with student *t* test, or Wilcoxon rank sum test, or Chi-squared test.

Abbreviations: FVC, forced vital capacity; FEV_1_, forced expiratory volume in 1 s; MMEF, maximal mid-expiratory flow; *D*_LCO_, diffusing capacity of the lung for carbon monoxide; *D*_LCO_/*V*_A_, *D*_LCO_ corrected for alveolar volume; TLC, total lung capacity; RV, residual volume; GGO, ground-glass opacity.

**Table S4. Comparison of CT finding according to diffusion capacity of the lung for carbon monoxide**

| **Variables** | **All patients** | ***D*_LCO_ ≥ 80%** | ***D*_LCO_ < 80%** | ***P* value *** |
| --- | --- | --- | --- | --- |
| **CT scan, N** | 106 | 68 | 38 |  |
| Involvement of the lesions, N (%) |  |  |  | 0.352 |
| No involvement | 40 (37.7) | 29 (42.6) | 11 (28.9) |  |
| Single lobe | 30 (28.3) | 17 (25.0) | 13 (34.2) |  |
| Bilateral multilobe | 36 (34.0) | 22 (32.4) | 14 (36.8) |  |
| Findings, N (%) |  |  |  |  |
| Reticulation | 44 (41.5) | 25 (36.8) | 19 (50.0) | 0.262 |
| GGO | 38 (35.8) | 22 (32.4) | 16 (42.1) | 0.428 |
| Bronchiectasis | 13 (12.3) | 7 (10.3) | 6 (15.8) | 0.604 |
| Parenchymal band | 7 (6.6) | 5 (7.4) | 2 (5.3) | 0.994 |
| Air trapping | 4 (3.8) | 1 (1.5) | 3 (7.9) | 0.257 |
| Crazy-paving pattern | 7 (6.6) | 3 (4.4) | 4 (10.5) | 0.419 |
| Consolidation | 5 (4.7) | 4 (5.9) | 1 (2.6) | 0.780 |
| Honeycombing | 1 (0.9) | 0 (0.0) | 1 (2.6) | 0.767 |
| No. of lobes involved, median (IQR) | 1 (0 -2) | 1 (0 -2) | 1 (0 - 3) | 0.141 |
| Total CT score, mean (SD) | 2.33 (3.44) | 1.85 (2.63) | 3.18 (4.45) | 0.056 |

* *P* values were calculated with student *t* test, or Wilcoxon rank sum test, or Chi-squared test.

Abbreviations: *D*_LCO_, diffusing capacity of the lung for carbon monoxide; GGO, ground-glass opacity.

**Table S5. The kidney function in recovery patients** **with COVID-19 at one-year follow-up**

| **Variables** | **All patients** | **Non-severe patients** | **Severe patients** | ***P* value** * |
| --- | --- | --- | --- | --- |
| **Kidney function at admission, N** | 207 | 162 | 45 |  |
| Blood detection |  |  |  |  |
| eGFR, mean (SD), mL/min/1.73 m^2^ | 101.77 (17.92) | 105.06 (15.35) | 89.92 (21.40) | **<0.001** |
| eGFR categories, mL/min/1.73 m^2^, N (%) |  |  |  |  |
| G1: ≥ 90 | 164 (79.2) | 136 (84.0) | 28 (62.2) | **<0.001** |
| G2: 60-89 | 38 (18.4) | 26 (16.0) | 12 (26.7) |  |
| G3a: 45-59 | 4 (1.9) | 0 (0.0) | 4 (8.9) |  |
| G3b: 30-44 | 1 (0.5) | 0 (0.0) | 1 (2.2) |  |
| **Kidney function at one-year, N** | 214 | 168 | 46 |  |
| Blood detection |  |  |  |  |
| BUN, mean (SD), mmol/L | 4.82 (1.38) | 4.65 (1.21) | 5.43 (1.74) | **0.001** |
| eGFR, mean (SD), mL/min/1.73 m^2^ | 98.06 (17.97) | 100.72 (15.92) | 88.36 (21.57) | **<0.001** |
| eGFR categories, mL/min/1.73 m^2^, N (%) |  |  |  | **0.001** |
| G1: ≥ 90 | 153 (71.5) | 128 (76.2) | 25 (54.3) |  |
| G2: 60-89 | 54 (25.2) | 38 (22.6) | 16 (34.8) |  |
| G3a: 45-59 | 5 (2.3) | 2 (1.2) | 3 (6.5) |  |
| G3b: 30-44 | 2 (0.9) | 0 (0.0) | 2 (4.3) |  |
| Urine detection ^a^ |  |  |  |  |
| PRO, N (%) | 36 (19.5) | 22 (15.1) | 14 (35.9) | **0.007** |
| Urinary micro-protein detection ^b^ |  |  |  |  |
| A1M, mean (SD), mg/L | 9.78 (9.51) | 8.32 (6.33) | 15.31 (15.78) | **<0.001** |
| B2M, mean (SD), mg/L | 0.27 (0.38) | 0.21 (0.16) | 0.47 (0.76) | **<0.001** |
| RBP, mean (SD), mg/L | 0.71 (0.21) | 0.69 (0.10) | 0.80 (0.41) | **0.007** |
| KAP, mean (SD), mg/L | 11.24 (10.07) | 9.91 (6.08) | 16.29 (17.94) | **0.001** |
| LAM, mean (SD), mg/L | 4.67 (2.57) | 4.38 (1.64) | 5.80 (4.56) | **0.004** |

* *P* values were calculated with student *t* test or Chi-squared test. ^a^ 29 missing, ^b^ 46 missing.

Abbreviations: BUN, blood urea nitrogen; eGFR, estimated glomerular ﬁltration rate; PRO, urine protein; A1M, α1-microglobulin; B2M, β2-microglobulin; RBP, retinol-binding protein; KAP, kappa free light chain; LAM, lambda light chain.

**Table S6. Laboratory biomarkers in recovery patients with COVID-19 at one-year follow-up**

| **Disease severity** | **All patients** | **Non-severe** | **Severe** | ***P* value** * |
| --- | --- | --- | --- | --- |
| N | 214 | 168 | 46 |  |
| LDH, mean (SD), U/L | 173.29 (33.38) | 169.46 (27.75) | 187.51 (46.66) | **0.001** |
| CRP, mean (SD), mg/L | 1.51 (1.91) | 1.30 (1.34) | 2.32 (3.20) | **0.003** |
| IL6, mean (SD), pg/mL | 2.45 (4.28) | 2.12 (2.35) | 3.70 (8.14) | **0.029** |
| D-dimer, mean (SD), µg/mL | 0.31 (0.16) | 0.29 (0.09) | 0.37 (0.28) | **0.003** |
| **Diffusion impairment** | **All patients** | ***D*_LCO_ ≥ 80%** | ***D*_LCO_ < 80%** | ***P* value** * |
| N | 110 | 71 | 39 |  |
| LDH, mean (SD), U/L | 173.55 (35.10) | 172.47 (31.67) | 175.53 (41.05) | 0.668 |
| CRP, mean (SD), mg/L | 1.69 (2.43) | 1.31 (1.65) | 2.36 (3.34) | **0.043** |
| IL6, mean (SD), pg/mL | 2.69 (5.38) | 1.99 (1.54) | 3.98 (8.75) | 0.066 |
| D-dimer, mean (SD), µg/mL | 0.30 (0.12) | 0.28 (0.08) | 0.33 (0.16) | **0.026** |
| **CT scan** | **All patients** | **No involvement** | **Involvement** | ***P* value** * |
| N | 197 | 74 | 123 |  |
| LDH, mean (SD), U/L | 172.53 (33.63) | 162.29 (24.78) | 178.66 (36.70) | **0.001** |
| CRP, mean (SD), mg/L | 1.53 (1.95) | 1.14 (1.19) | 1.75 (2.25) | **0.046** |
| IL6, mean (SD), pg/mL | 2.48 (4.45) | 1.80 (0.80) | 2.89 (5.56) | 0.098 |
| D-dimer, mean (SD), µg/mL | 0.31 (0.16) | 0.29 (0.10) | 0.31 (0.19) | 0.426 |
| **eGFR** | **All patients** | **eGFR ≥ 90** | **eGFR < 90** | ***P* value** * |
| N | 214 | 153 | 61 |  |
| LDH, mean (SD), U/L | 173.29 (33.38) | 169.68 (31.72) | 182.43 (35.94) | **0.012** |
| CRP, mean (SD), mg/L | 1.51 (1.91) | 1.44 (1.83) | 1.68 (2.09) | 0.441 |
| IL6, mean (SD), pg/mL | 2.45 (4.28) | 2.24 (2.79) | 2.96 (6.73) | 0.272 |
| D-dimer, mean (SD), µg/mL | 0.31 (0.16) | 0.29 (0.10) | 0.35 (0.24) | **0.012** |

* *P* values were calculated with student *t* test.

Abbreviations: LDH, lactate dehydrogenase; CRP, C-reactive protein; IL6, Interleukin 6; *D*_LCO_, diffusing capacity of the lung for carbon monoxide; eGFR, estimated glomerular ﬁltration rate.

**Table S7. SARS-CoV-2 RBD-specific antibody levels and seropositive rate in recovery patients with COVID-19 at one-year follow-up**

| **Variables** | **All patients** | **Non-severe patients** | **Severe patients** | ***P* value** * |
| --- | --- | --- | --- | --- |
| N | 222 | 173 | 49 |  |
| IgA, median (IQR), COI | 1.20 (0.60, 2.28) | 1.14 (0.55, 2.08) | 1.32 (0.88, 3.28) | **0.009** |
| IgA ≥1.0 COI, N (%) | 126 (56.8) | 91 (52.6) | 35 (71.4) | **0.029** |
| IgG, median (IQR), COI | 10.10 (5.76, 14.02) | 8.61 (5.10, 12.72) | 14.04 (11.63, 16.36) | **<0.001** |
| IgG ≥1.0 COI, N (%) | 213 (95.9) | 164 (94.8) | 49 (100.0) | 0.223 |
| IgM, median (IQR), COI | 0.32 (0.12, 0.63) | 0.28 (0.11, 0.55) | 0.38 (0.15, 0.83) | 0.054 |
| IgM ≥1.0 COI, N (%) | 34 (15.4) | 24 (14.0) | 10 (20.4) | 0.379 |

* *P* values were calculated with Wilcoxon signed-rank test or Chi-squared test.

Abbreviations: COI, Cut-off index.

**Table S8. Risk factors associated with psychological symptoms, diffusion impairment, radiological abnormalities, decreased eGFR, and decreased IgG.**

|  | **Univariable analysis** | |  | **Multivariate analysis *** | |
| --- | --- | --- | --- | --- | --- |
|  | **OR (95% CI)** | **P value** |  | **OR (95% CI)** | **P value** |
| **Psychological symptoms** |  |  |  |  |  |
| Anxiety |  |  |  |  |  |
| Age, ≥ 50 y vs. < 50 y | 2.90 (1.03-8.86) | **0.049** |  | 3.13 (1.05-10.01) | **0.044** |
| Depression |  |  |  |  |  |
| Age, ≥ 50 y vs. < 50 y | 5.11 (1.99-14.87) | **0.001** |  | 5.86 (2.17-17.71) | **0.001** |
| PTSS |  |  |  |  |  |
| Age, ≥ 50 y vs. < 50 y | 2.64 (1.04-7.07) | **0.044** |  | 3.09 (1.16-8.65) | **0.026** |
| Sleep difficulties |  |  |  |  |  |
| Age, ≥ 50 y vs. < 50 y | 2.23 (1.16-4.29) | **0.016** |  | 2.56 (1.28-5.19) | **0.008** |
| **Diffusion impairment** |  |  |  |  |  |
| Sex, Female vs. Male | 4.00 (1.77-9.55) | **0.001** |  | 4.73 (1.98-12.24) | **0.001** |
| Severity, Severe vs. Non-severe | 2.22 (0.91-5.48) | 0.080 |  | 2.97 (1.02-9.04) | **0.049** |
| **Radiological abnormalities** |  |  |  |  |  |
| Age, ≥ 50 y vs. < 50 y | 5.00 (2.58-10.28) | **<0.001** |  | 4.30 (2.13-9.16) | **<0.001** |
| Severity, Severe vs. Non-severe | 4.44 (1.98-11.36) | **0.001** |  | 2.75 (1.15-7.34) | **0.030** |
| **Decreased eGFR** |  |  |  |  |  |
| Age, ≥ 50 y vs. < 50 y | 14.29 (7.04-31.03) | **<0.001** |  | 18.44 (8.34-44.65) | **<0.001** |
| Sex, Male vs. Female | 1.60 (0.88-2.92) | 0.126 |  | 3.00 (1.39-6.82) | **0.006** |
| **Decreased IgG** |  |  |  |  |  |
| Severity, Non-severe vs. Severe | 5.51 (1.84-23.82) | **0.007** |  | 4.68 (1.47-20.93) | **0.019** |

* In multivariable model, age, sex, and disease severity were adjusted.

Abbreviations: PTSS, post-traumatic stress symptoms; eGFR, estimated glomerular ﬁltration rate.
